# Supplementary material for: The mRNA binding-mediated self-regulatory function of small heat shock protein IbpA in γ-proteobacteria is conferred by a conserved arginine
Source: J Biol Chem. 2023 Jul 28;299(9):105108. doi: 10.1016/j.jbc.2023.105108 (PMC10474464; doi:10.1016/j.jbc.2023.105108)
Supplement: Supporting Figures S1–S10 and Tables S1 and S2 [file mmc1.pdf]

Supporting Information

The mRNA binding-mediated self-regulatory function of small heat shock protein IbpA in γ-proteobacteria is conferred by a conserved arginine

Yajie Cheng, Tsukumi Miwa and Hideki Taguchi*

*Correspondence author: Hideki Taguchi
Email: taguchi@bio.titech.ac.jp

This PDF file includes:

Figures S1 to S10
Tables S1, S2

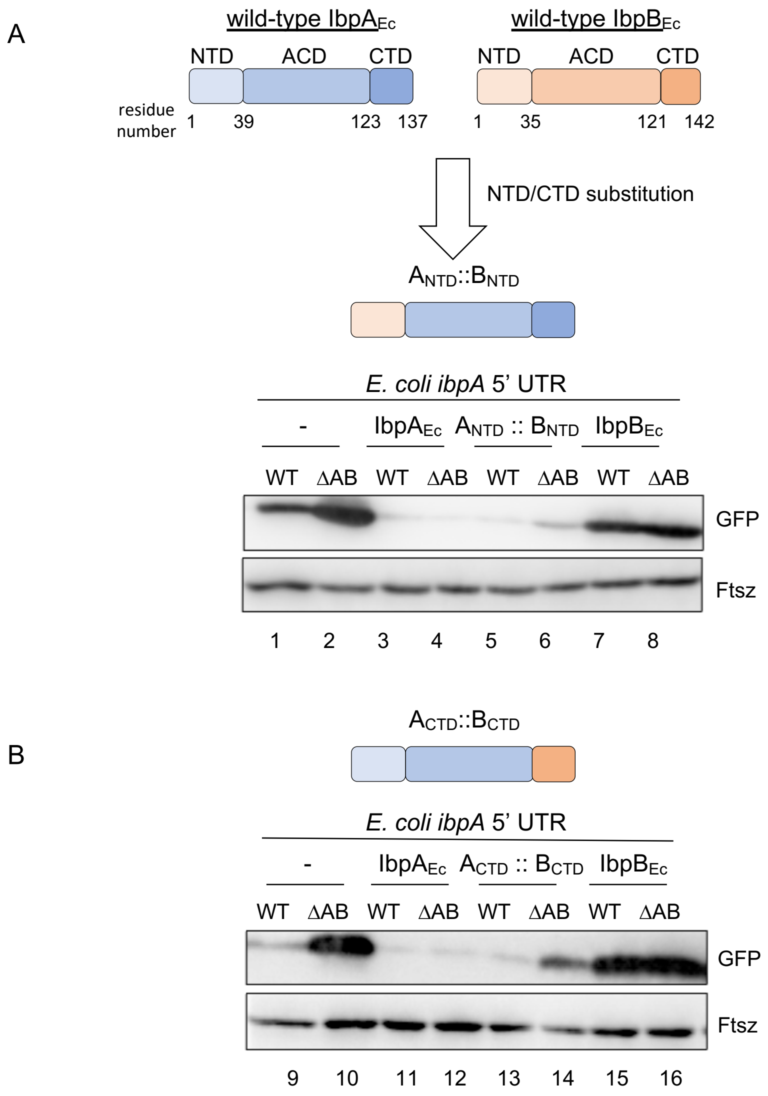

Fig. S1. Effects of IbpA$_{Ec}$/IbpB$_{Ec}$ chimeras in the N-terminal domain (NTD) or C-terminal domain (CTD) on the IbpA$_{Ec}$-mediated translation suppression.
Upper: a schematic representation of IbpA$_{Ec}$/IbpB$_{Ec}$ chimeras in NTD (*A*) or CTD (*B*), where IbpA$_{Ec}$ and IbpB$_{Ec}$ are denoted by blue and orange, respectively. *Lower*: Western blotting analysis to evaluate the effects of the NTD (*A*) and CTD (*B*) chimeras on the level of the GFP reporter translation in *E. coli* BW25113 strains (WT: BW25113 wild-type strain; ΔAB: *ibpAB* operon-deleted BW25114 strain). The constitutive expression level of FtsZ is shown as a control. Anti-GFP and anti-FtsZ antibodies were used for the detection.

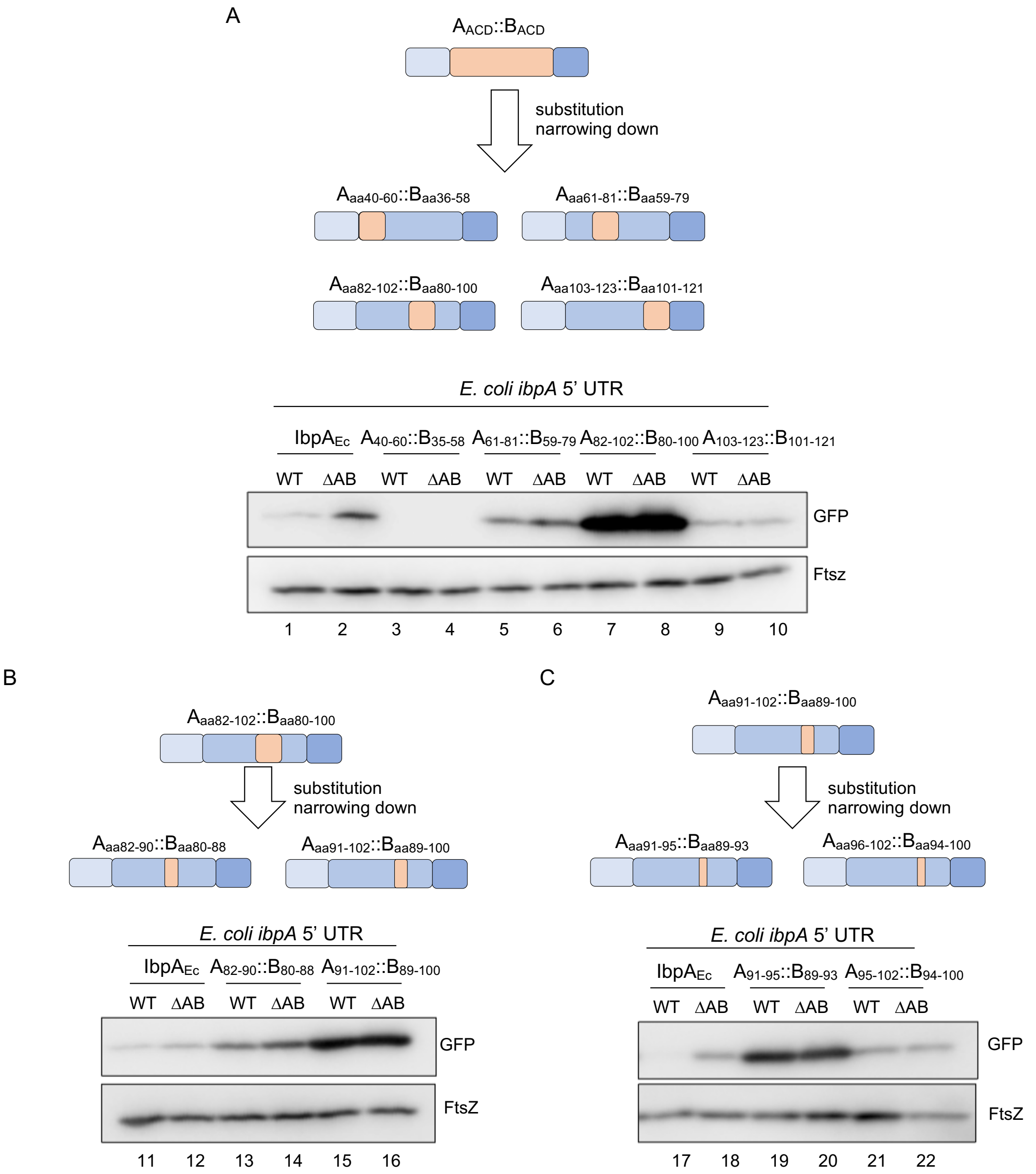

Fig. S2. Systematic mutagenesis introduced in the IbpA$_{Ec}$-ACD to identify a region crucial for IbpA$_{Ec}$-mediated translation suppression.

Following Fig. S1, IbpA$_{Ec}$/IbpB$_{Ec}$ chimeras further subdivided within the ACD are analyzed. The reporter assay method, including western blotting, is the same as that used in Fig. 1C, D and Fig. S1. (*A*) The ACD of IbpA$_{Ec}$ was subdivided into four parts, and the corresponding portions of IbpB$_{Ec}$ were individually substituted for each part. The A_{82-102}::B_{80-100} chimera (IbpA$_{Ec}$ residues from 82 to 102 were replaced with IbpB$_{Ec}$ residues from 80 to 100) was found to be suppression-inactive compared to the other three chimeras and the wild-type IbpA$_{Ec}$. (*B*, *C*) The substitution regions of the suppression-defect chimera A_{82-102}::B_{80-100} were further narrowed down until the IbpA$_{Ec}$ 91-95 region was identified as being critical for translation suppression.

```
                       E.coli IbpA   -M--------------RNFDL-SPLYRS-AIG---------------------------- 15
        Agrobacterium tumefaciens IbpA   -M--------------RHVDF-SPLYRS-TVG---------------------------- 15
          Caulobacter vibrioides IbpA   MT--------------RTILFDSP---F-LLG---------------------------- 14
              Rhizobium indicum IbpA   MS--------------RITPFASP---L-LLG---------------------------- 14
              Bacillus subtilis IbpA   -----------MFEWNKYFPFHNQFSKEALK----------KADPKEVETYVNRVMESVF 39
     Lactobacillus acidophilus Hsp20   ----------------------------------------------MAND----MMNR-- 8
         Clostridium butyricum Hsp20   MFGMFPFGMGNMISFTSFTSFTST--KDGINGFNVTNGYSGFFDPNQANN-FNQNMNNMN 57

                                                                           34
                       E.coli IbpA   ----------------------------FDRLFNHLENN-QSQSNGGYPPYNVELVD--- 43
        Agrobacterium tumefaciens IbpA   ----------------------------FDRLFSMLDGLGQPEQAQSYPPYNIERTG--- 44
          Caulobacter vibrioides IbpA   ----------------------------FEHTRDLIERA-AKAASESYPPYNVEQAE--- 42
              Rhizobium indicum IbpA   ----------------------------FDAMEKTLERI-SK-ASDGYPPYNIERIAADT 44
              Bacillus subtilis IbpA   GSDYAA---------------------QFPF-RDPLPQKEQP-AKSAKPDIDIFET---- 72
     Lactobacillus acidophilus Hsp20   -----------------RNDMMDAMNDWFGFPRNFFD--DSE-I-ENIMQSDVAET---- 43
         Clostridium butyricum Hsp20   GMNLLDHIQSAVTSVLNNVDIEKLAEEYYTAISDTIK--ENS-IEDDCDFIDFERN---- 110
                                                                   :    . :               :.

                       E.coli IbpA   --ENHYRIAIAVAGFAESELEITA----------------------------------QD 67
        Agrobacterium tumefaciens IbpA   --ENTYRITMAVAGFDENELSIES----------------------------------RA 68
          Caulobacter vibrioides IbpA   --HGGVRITLAVAGFSPEQLQVTV----------------------------------EG 66
              Rhizobium indicum IbpA   GAPERLRITLAVAGFSEEELDVSI----------------------------------EE 70
              Bacillus subtilis IbpA   --TDHVFVKVPISESQLEQLKIKHTSHALMIENFPNLDHPKKISLPCLVKRKGTKAVYKD 130
     Lactobacillus acidophilus Hsp20   --DKDYVVKIDMPGMNKDD----------------------------------IKLNYKD 67
         Clostridium butyricum Hsp20   --DDMYILRIDLNGIDLRE----------------------------------LSIRYDP 134
                                              : : :      :

                                                          93
                       E.coli IbpA   NLLVVKGAHA--D--EQKERTYLYQGIAERNFERKFQLAENIHV-----RGANLVNGLLY 118
        Agrobacterium tumefaciens IbpA   NALTVKAEKA--TDEKTTEGEFLYRGIATRAFERRFQLADHVEV-----QTASLKNGLLH 121
          Caulobacter vibrioides IbpA   GQLVVAGKRDSADARSEAERAFLHRGIAARGFVRTFVLAEGMEV-----TAATLEHGLLH 121
              Rhizobium indicum IbpA   NQLVIRGRQV-----EQGERDYLYRGIAARQFQRTFVLADGMQV-----LGAGLKNGLLS 120
              Bacillus subtilis IbpA   GLLEVMFQKQQDY--NMSEVE----IIR-------------------------------- 152
     Lactobacillus acidophilus Hsp20   GVLSVAGTRKAFK--DTNDKE--RNIIHKERSEGSISRSYRLPNVVANEIHAKYDNGVLT 123
         Clostridium butyricum Hsp20   GILDINLKRSEYD--NNSYRGYTNNIVKKK-YNTSF---DNIEEIDTDRVLKSIDNGIFT 188
                                       . * :   :      .          :

                       E.coli IbpA   IDLERVIPEAKKPRRIEIN---------------------- 137
        Agrobacterium tumefaciens IbpA   IDLVRNIPEAMKPRRIAISSDSAD--APKTIEAQISPAQVN 160
          Caulobacter vibrioides IbpA   IDLARPAPERLVK-KIPIRSAG------------------- 142
              Rhizobium indicum IbpA   VDLIRPEPARMVK-KINISVSQ------------------- 141
              Bacillus subtilis IbpA   ----------------------------------------- 152
     Lactobacillus acidophilus Hsp20   ITLPKQTAGDDDNSIQI-D---------------------- 141
         Clostridium butyricum Hsp20   MRMPKKYALDSASKIIEVENYTVDNENDKVIKKM------- 222
```

Fig. S3. Alignment of *E. coli* IbpA with typical α-proteobacterial and gram-positive bacterial IbpAs.

α-proteobacterial and gram-positive bacterial IbpAs (or annotated Hsp20) are colored green and orange respectively. Y34 and R93 residues are conserved in **α**-proteobacteria but not in gram-positive bacteria, suggesting the conservation of IbpA self-regulation in **α**-proteobacteria .

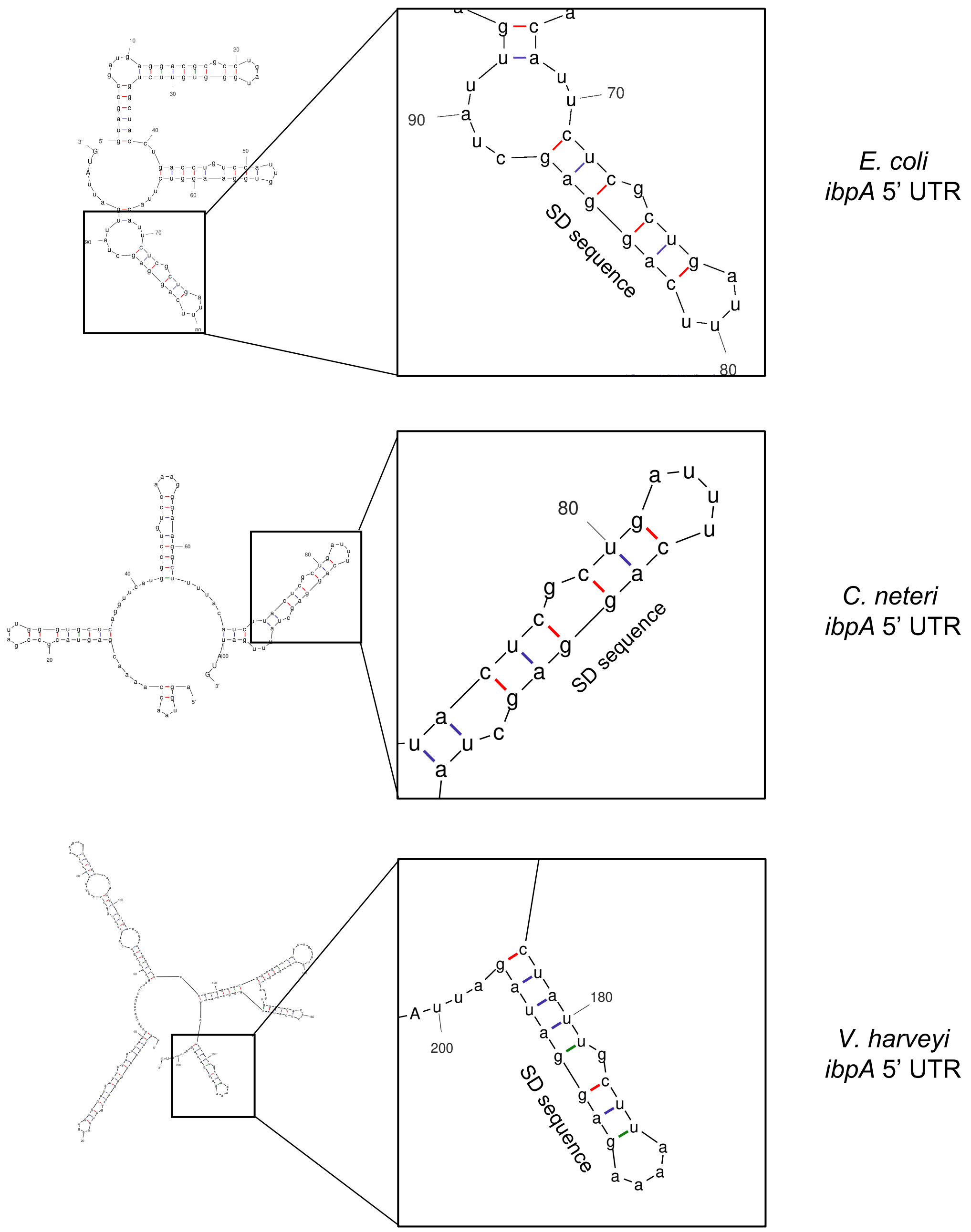

Fig. S4. Prediction of RNAT-like structures in the 5’ UTR of bacterial *ibpA* mRNAs.

The secondary structures of the 5’ UTR mRNAs of $ibpA_{Ec}$, $ibpA_{Cn}$, and $ibpA_{Vh}$, whose sequences are listed in Table S1, were predicted using UNAFold (http://www.unafold.org/). The Shine-Dalgarno sequences are highlighted.

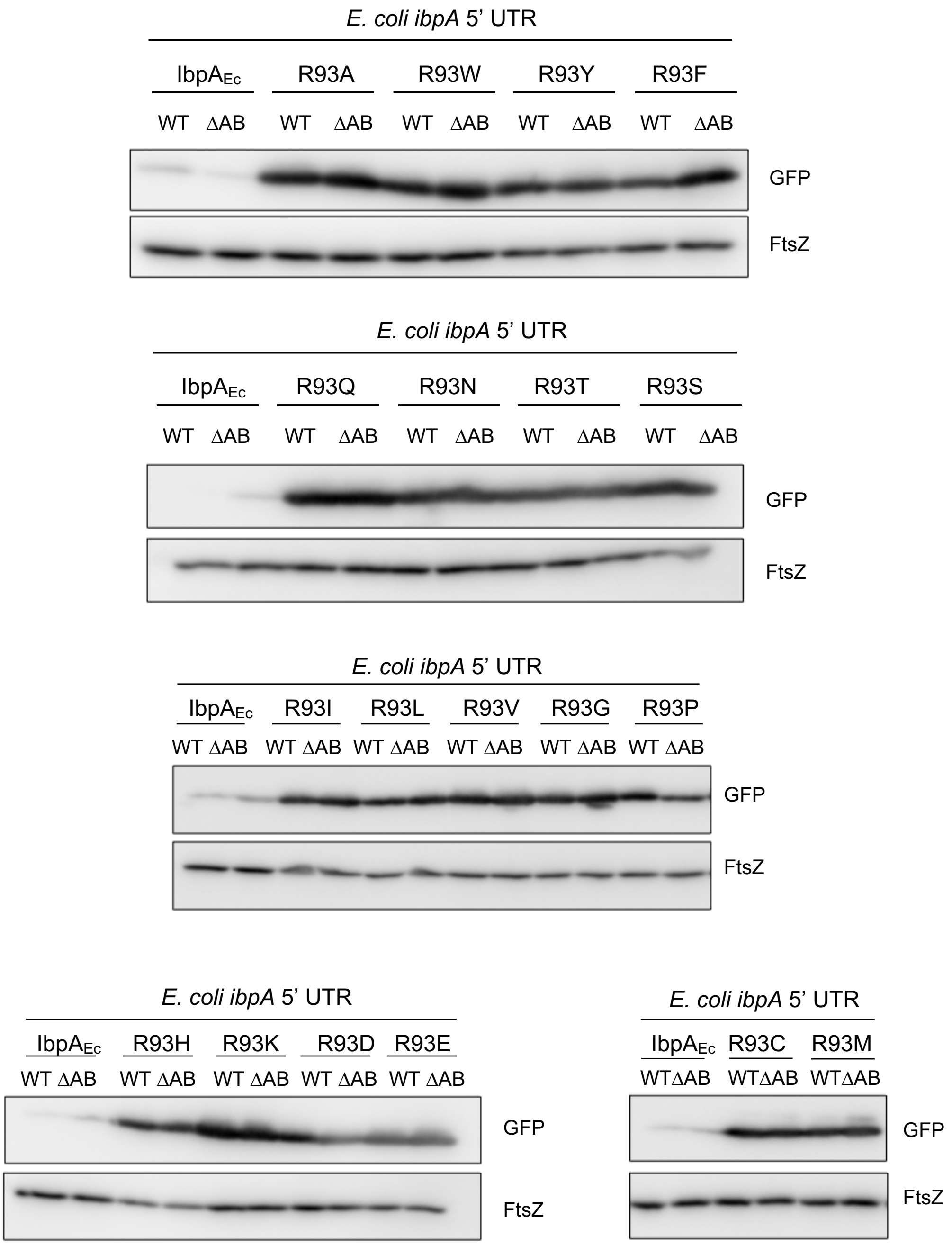

Fig. S5. Mutations of the IbpA$_{Ec}$-R93 residue to other 19 amino acids.

The arginine 93 in IbpA$_{Ec}$ was individually mutated to 19 other amino acids. The reporter assay method, including western blotting, is the same as that used in Fig. 1C, D and Fig. S1 and S2.

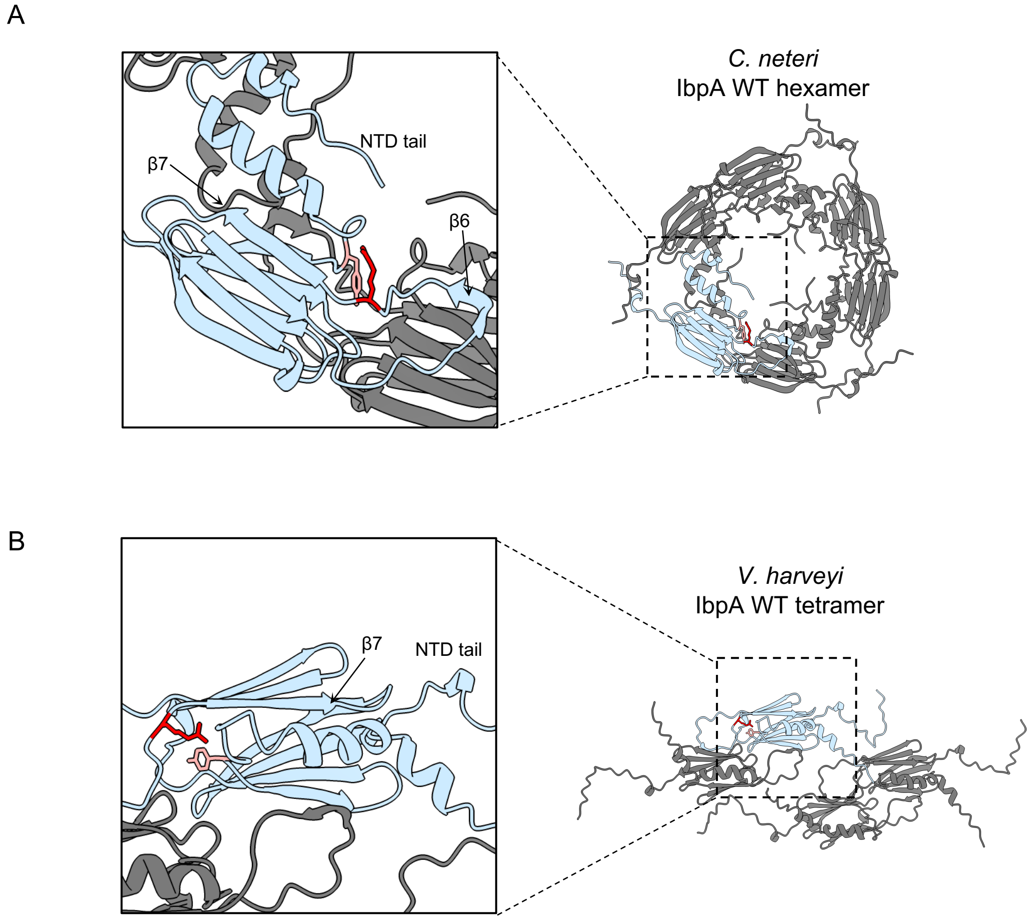

Fig. S6. Potential interaction between conserved Y34 and R93 based on AlphaFold2-predicted IbpA structures.

AlphaFold2-predicted structures of IbpA$_{Cn}$-WT hexamer (*A*) and IbpA$_{Vh}$-WT tetramer (*B*) using MMseqs2 (33), are shown. One of the subunits is colored light blue for clarity. Enlarged images indicate that Y34 and the Arg residues corresponding to IbpA$_{Ec}$-R93 (IbpA$_{Cn}$-R93 and IbpA$_{Vh}$-R94) are colored pink and red, respectively.

A

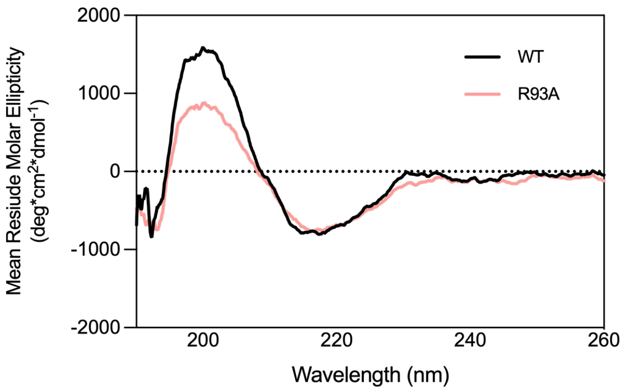

B

%	α-Helix	Anti-parallel	Parallel	β-Turn	random coil
WT	0	42	0	13.3	44.7
R93A	0	41.9	0	13.2	44.9

Fig. S7. Circular Dichroism (CD) spectra of purified IbpA proteins.
(*A*) The far-UV CD spectra of $IbpA_{Ec}$-WT, R93A (1.0 mg/ml) were measured using a JASCO J-820 spectropolarimeter (Japan) at 20 °C in a buffer containing 50 mM sodium phosphate pH 7.6. The measurement was performed using a 1 mm path-length cell, and each spectrum was an average of 10 accumulations. (*B*) The estimation of the secondary structure contents of IbpAs was calculated using BeStSel https://bestsel.elte.hu/index.php.

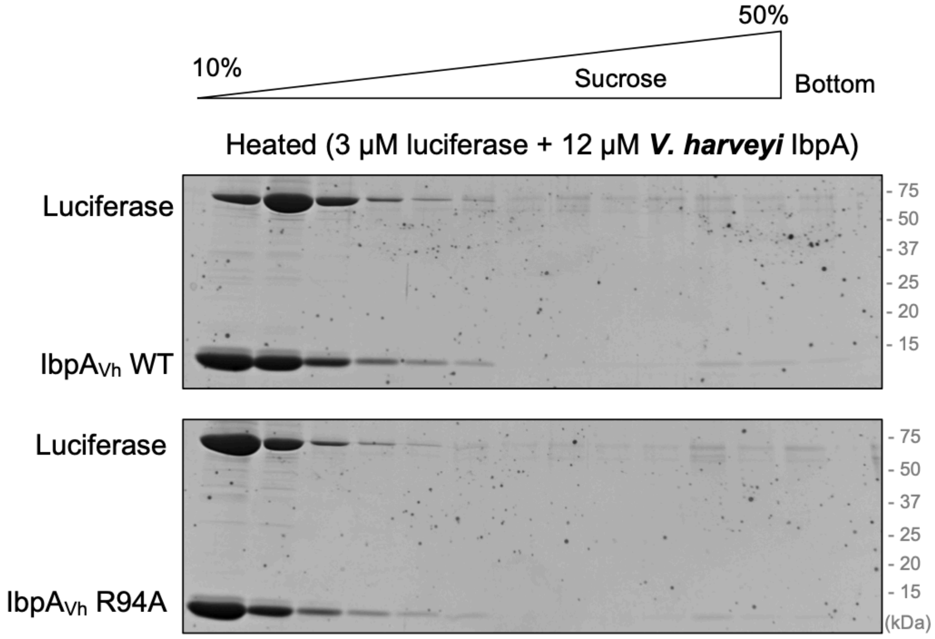

Fig S8. IbpA$_{Vh}$ R94A shows similar chaperone activity compared to IbpA$_{Vh}$ WT. The interaction between IbpA$_{Vh}$ and denatured luciferase was analyzed using SDG centrifugation. Luciferase was thermally denatured in the presence of IbpA$_{Vh}$ WT or R93 mutant, and then the mixtures were applied to 10%-50% (w/v) sucrose gradient solutions, followed by SDS-PAGE and CBB staining.

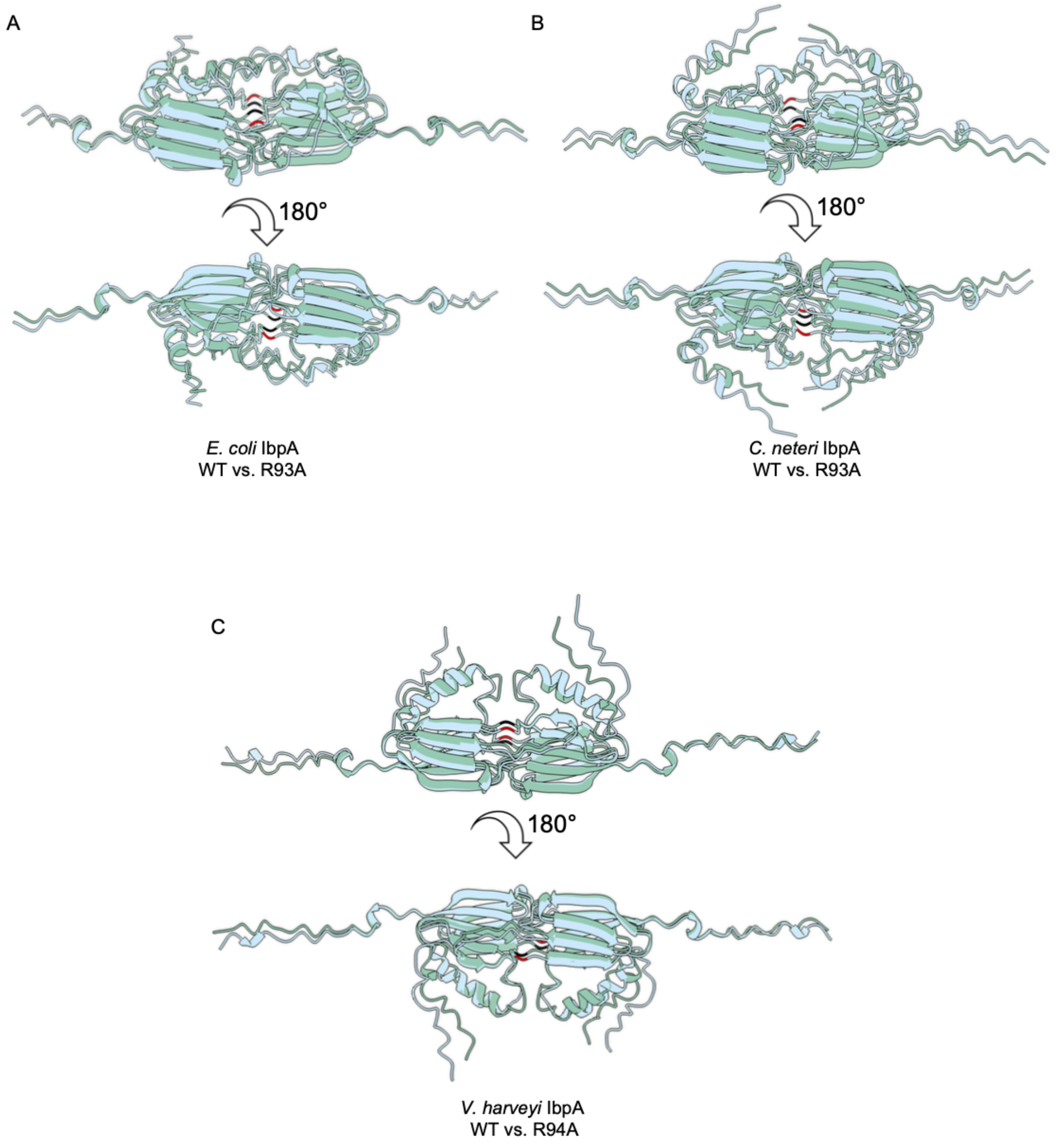

Fig. S9. Predicted dimer structures of IbpA-WT and the R93 mutants.

Predicted dimer structures of IbpA WT (*blue*) and the R93 mutants (*green*) in *E. coli* (*A*), *C. neteri* (*B*), and *V. harveyi* (*C*). The prediction was carried out using AlphaFold2 using MMseqs2 (33). The Arg93 (and the equivalent Arg residues) in the WT and Ala93 (and the equivalent Ala residues) in the R93A mutants are colored red and black, respectively.

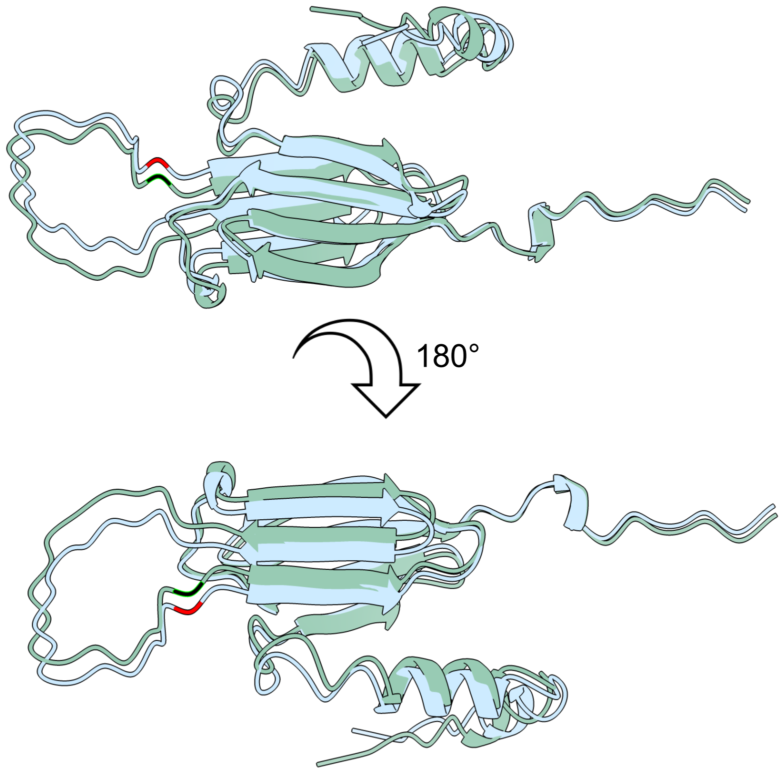

Fig. S10. Predicted monomer structures of IbpA$_{Ec}$-WT and R93A.
Predicted monomer structures of IbpA$_{Ec}$-WT (*blue*) and R93A (*green*) were obtained using AlphaFold2 with MMseqs2 (33). The R93 in WT and A93 in the R93A mutant are colored red and black, respectively.

Table S1. 5’ UTR sequences of IbpAs examined in this study.

**Name**	**Sequence**
$ibpA_{Ec}$ 5’ UTR	gtagccgatgaggacgcgcctgatgggtgttctggctacctgacctgtccattg<br>tggaaggtcttacattctcgctgatttcaggagctattgattATG
$ibpA_{Cn}$ 5’ UTR	aggtaaccaaaacgagtacgccgattgggtgctcaggttcatggcctgtccaaa<br>gggaaggcttttacatcttactcgctgatttcaggagctatttgatATG
$ibpA_{Vh}$ 5’ UTR	cgctaaacgagatcgctcaagagaggatactcagtattagcacacaacttgaccc<br>tagattcgacatcatgtccccgcacctaaagagtaggtcagaggaatgaatcatcgg<br>atcccttagctcggcgttttcttgcataaatgcgccacgtggatgaccctcatctgggcta<br>agactattgcttaaaagaggatagattATG

Table S2. Primers used in this study.

Primer name	Sequence
pCA_ibpB_Fw	GAGGAGAAATTAACTATGCGTAACTTCGATTTATCCCCACTG
pCA_ibpB_Rv	GCTGCAGGTCGACCCTTAGCTATTTAACGCGGACGTTCGC
ibpA-N::ibpB-N_Rv	GGTTTTCGTCTACCAGTTCgtacggcgggaagctctggctttc
ibpB-N_ibpA_alpha_Fw	gagcttcccgccgtacGAACTGGTAGACGAAAACCATTACCGC
ibpA_alpha_Rv	GCGTTCGAGATCGATATACAGCAAACCATTTACC
ibpA_alpha_ibpB_Cd_Fw	TGTATATCGATCTCGAACGCAATGAGCCTGAACCCATCGCAG
ibpB-C::ibpA-C_Fw	CATATTGATTTAATTCGTGTGATTCCGGAAGCGAAAAAACCG
ibpB-C::ibpA-C_Rv	CGCTTCCGGAATCACACGAATTAAATCAATATGCAGTAAACCG
ibpB-N::ibpA-N_Rv	cgtcgcttttctcaatgttAACGTTATACGGAGGGTAGCCGCC
ibpA-N_ibpB_alpha_Fw	CCCTCCGTATAACGTTaacattgagaaaagcgacgataacc
ibpB-C::ibpA-C_Fw	CATATTGATTTAATTCGTGTGATTCCGGAAGCGAAAAAACCG
ibpB-C::ibpA-C_Rv	CGCTTCCGGAATCACACGAATTAAATCAATATGCAGTAAACCG
pCA_ibpA_alpha1/21::B_ins_Fw	CGGCTACCCTCCGTATAACGTTgagaaaagcgacgataaccactaccgc
pCA_ibpA_alpha1/21::B_ins_Rv	CTGGGCGGTAATTTCCAGatcttcctgacggaaacctgccagcg
pCA_ibpA_alpha1/21::B_vec_Fw	GGAAATTACCGCCCAGGATAATCTGCTGGTGGTGAAAGGTGCTCACGC
pCA_ibpA_alpha1/21::B_vec_Rv	TACGGAGGGTAGCCGCCATTACTCTGGCTCTGGTTGTTTTCTAAGTG
pCA_ibpA_alpha22/42::B_ins_Fw	GTGGCTGGTTTTGCTGAGAGCGAAttagagattcaactggaaggtacg
pCA_ibpA_alpha22/42::B_ins_Rv	GGTACAGATAGGTGCGCTCttcttttggctgctccggcgtg
pCA_ibpA_alpha22/42::B_vec_Fw	GCGCACCTATCTGTACCAGGGCATCGCTGAACGCAACTTTGAAC
pCA_ibpA_alpha22/42::B_vec_Rv	CAGCAAAACCAGCCACAGCGATAGCAATGCGGTAATGGTTTTCGTC
pCA_ibpA_alpha43/63::B_ins_Fw	CTCACGCCGACGAACAAAAAgagaaaaaatggctgcatcaagggc
pCA_ibpA_alpha43/63::B_ins_Rv	CAGGTTAGCACCACGAACATGAATGTTCTCagccagcgtaaagctcagg
pCA_ibpA_alpha43/63::B_vec_Fw	GTTCGTGGTGCTAACCTGGTAAATGGTTTGCTGTATATCGATCTCGAAC
pCA_ibpA_alpha43/63::B_vec_Rv	GTTCGTCGGCGTGAGCACCTTTCACCACCAGCAGATTATCCTGGG
pCA_ibpA_alpha64/84::B_ins_Fw	GAACGCAAATTCCAGTTAGCTgaaaatatggaagtctctggcgcaacc
pCA_ibpA_alpha64/84::B_ins_Rv	CGCTTCCGGAATCACacgaattaaatcaatatgcagtaaaccgtttacg
pCA_ibpA_alpha64/84::B_vec_Fw	gtGTGATTCCGGAAGCGAAAAAACCGCGCCGTATCGAAATCAACtaaGG
pCA_ibpA_alpha64/84::B_vec_Rv	cAGCTAACTGGAATTTGCGTTCAAAGTTGCGTTCAGCGATGCCCTGG
pCA_ibpA_alpha43/51::B_Fw	gagaaaaaatggctgcatcaagggcttGCTGAACGCAACTTTGAACGC
pCA_ibpA_alpha43/51::B_Rv	CaagcccttgatgcagccattttttctcTTTTTGTTCGTCGGCGTGAG
pCA_ibpA_alpha52/63::B_ins_Fw	CTATCTGTACCAGGGCATCatgaatcagccatttagcctg
pCA_ibpA_alpha52/63::B_ins_Rv	CCACGAACATGAATGTTCTCagccagcgtaaagctcag
pCA_ibpA_alpha52/63::B_vec_Fw	GAGAACATTCATGTTCGTGGTG
pCA_ibpA_alpha52/63::B_vec_Rv	GATGCCCTGGTACAGATAGG
pCA_ibpA_alpha52/56::B_Fw	CatgaatcagccatttGAACGCAAATTCCAGTTAGC
pCA_ibpA_alpha52/56::B_Rv	CaaatggctgattcatGATGCCCTGGTACAGATAGG
pCA_ibpA_alpha57/63::B_Fw	agcctgagctttacgctggctGAGAACATTCATGTTCGTGG
pCA_ibpA_alpha57/63::B_Rv	agccagcgtaaagctcaggctAAAGTTGCGTTCAGCGATG
pCA_ibpA_E92A_Fw	GGCATCGCTgcgCGCAAC
pCA_ibpA_E92A_Rv	GTTGCGgcgAGCGATGCC

pCA_ibpA_R93A_Fw	GGGCATCGCTGAAgcgAACTTTG
pCA_ibpA_R93A_Rv	CTGGAATTTGCGTTCAAAGTTcgcTTCAG
pCA_ibpA_N94A_Fw	GCTGAACGCgcgTTTGAACGC
pCA_ibpA_N94A_Rv	TGGAATTTGCGTTCAAAcgcGCG
pCA_ibpA_F95A_Fw	GCTGAACGCAACgcgGAACGC
pCA_ibpA_F95A_Rv	CTGGAATTTGCGTTCcgcGTTGCG
pCA_ibpA_R93K_Fw	GGGCATCGCTGAAaaaAACTTTGAACGC
pCA_ibpA_R93K_Rv	TTtttTTCAGCGATGCCCTGGTACAG
pCA_ibpA_R93N_Fw	GGCATCGCTGAAaacAACTTTGAACGC
pCA_ibpA_R93N_Rv	TTgttTTCAGCGATGCCCTGGTACAG
pCA_ibpA_R93W_Fw	GCATCGCTGAAtggAACTTTGAACGC
pCA_ibpA_R93W_Rv	TTccaTTCAGCGATGCCCTGGTACAG
pCA_ibpA_R93Y_Fw	CATCGCTGAAtatAACTTTGAACGCAAATTCCAG
pCA_ibpA_R93Y_Rv	TTataTTCAGCGATGCCCTGGTACAGATAGG
pCA_ibpA_R93F_Fw	GGCATCGCTGAAtttAACTTTGAACGCAAATTC
pCA_ibpA_R93F_Rv	TaaaTTCAGCGATGCCCTGGTACAGATAGG
pCA_ibpA_R93I_Fw	CTGTACCAGGGCATCGCTGAAattAACTTTG
pCA_ibpA_R93I_Rv	GTTCTCAGCTAACTGGAATTTGCGTTCAAAGTTaatTTC
pCA_ibpA_R93L_Fw	CCAGGGCATCGCTGAActgAACTTTG
pCA_ibpA_R93L_Rv	CAGCTAACTGGAATTTGCGTTCAAAGTTcagTTC
pCA_ibpA_R93V_Fw	CCAGGGCATCGCTGAAgtgAACTTTG
pCA_ibpA_R93V_Rv	CAGCTAACTGGAATTTGCGTTCAAAGTTcacTTC
pCA_ibpA_R93G_Fw	CCAGGGCATCGCTGAAggcAAC
pCA_ibpA_R93G_Rv	GAATTTGCGTTCAAAGTTgccTTCAGCG
pCA_ibpA_R93P_Fw	CCAGGGCATCGCTGAAccgAAC
pCA_ibpA_R93P_Rv	GGAATTTGCGTTCAAAGTTcggTTCAGC
pCA_ibpA_R93C_Fw	CAGGGCATCGCTGAAtgcAACTTTG
pCA_ibpA_R93C_Rv	GCTAACTGGAATTTGCGTTCAAAGTTgcaTTC
pCA_ibpA_R93M_Fw	ACCAGGGCATCGCTGAAatgAACTTTG
pCA_ibpA_R93M_Rv	CAGCTAACTGGAATTTGCGTTCAAAGTTcatTTC
pCA_ibpA_R93H_Fw	GTACCAGGGCATCGCTGAAcatAACTTTG
pCA_ibpA_R93H_Rv	CAGCTAACTGGAATTTGCGTTCAAAGTTatgTTC
pCA_ibpA_R93D_Fw	GTACCAGGGCATCGCTGAAgatAACTTTG
pCA_ibpA_R93D_Rv	CTCAGCTAACTGGAATTTGCGTTCAAAGTTatcTTC
pCA_ibpA_R93E_Fw	GAAgaaAACTTTGAACGCAAATTCCAGTTAGCTG
pCA_ibpA_R93E_Rv	CAAAGTTttcTTCAGCGATGCCCTGGTACAG
pCA_ibpA_R93Q_Fw	GAAcagAACTTTGAACGCAAATTCCAGTTAGCTG
pCA_ibpA_R93Q_Rv	CAAAGTTctgTTCAGCGATGCCCTGGTAC
pCA_ibpA_R93S_Fw	GAAagcAACTTTGAACGCAAATTCCAGTTAGC
pCA_ibpA_R93S_Rv	CAAAGTTgctTTCAGCGATGCCCTGG
pCA_ibpA_R93T_Fw	GAAaccAACTTTGAACGCAAATTCCAGTTAGC
pCA_ibpA_R93T_Rv	CAAAGTTggtTTCAGCGATGCCCTGGTAC
pBAD_sfGFP_Vec_Fw	ATGAGTAAAGGAGAAGAACTTTTCACTGGAGTTGTCC

pBAD_sfGFP_Vec_Rv	ATGGAGAAACAGTAGAGAGTTGCGATAAAAAGCGTCAG
pBAD_C.neteri ibpA 5' UTR_Fw	aggtaaccaaaacgagtacgccgattgg
pBAD_C.neteri_ibpA 5' UTR_Rv	gttttggttacctATGGAGAAACAGTAGAGAGTTGCGATAAAAAGCG
pBAD_V.harveyi_ibpA 5' UTR_Fw	AACTCTCTACTGTTTCTCCATcgctaaacgagatcgctc
pBAD_V.harveyi_ibpA 5'UTR_Rv	AAAGTTCTTCTCCTTTACTCATaatctatcctcttttaagcaatagtc
pCA_ibpA_C.neteri_R93A_Fw	AAgcgAACTTCGAGCGTAAATTTCAAC
pCA_ibpA_C.neteri_R93A_Rv	CGAAGTTcgcTTCCGCGATG
pCA_ibpA_V.harveyi_R94A_Fw	GAGgcgGATTTTGAACGTAAATTCCAAC
pCA_ibpA_V.harveyi_R94A_Rv	CAAAATCcgcCTCTGCAATGCC
pCA_ibpA_R97A_Fw	CGCTGAACGCAACTTTGAAgcgAAATTCC
pCA_ibpA_R97A_Rv	CGAACATGAATGTTCTCAGCTAACTGGAATTTcgcTTC
pCA_ibpA _R83A_Fw	GAGgcgACCTATCTGTACCAGGGC
pCA_ibpA_R83A_Rv	GGTcgcCTCTTTTTGTTCGTCGGC
pCA_ibpA _K98A_Fw	CGCgcgTTCCAGTTAGCTGAGAAC
pCA_ibpA_K98A_Rv	GAAcgcGCGTTCAAAGTTGCGTTCAG
pCA_ibpA_R97K_Fw	CGCAACTTTGAAaaaAAATTCCAGTTAGCTGAGAAC
pCA_ibpA_R97K_Rv	TTTtttTTCAAAGTTGCGTTCAGCGATGCC
pCA_ibpA_K98R_Fw	GAACGCcgcTTCCAGTTAGCTGAG
pCA_ibpA_K98R_Rv	gcgGCGTTCAAAGTTGCGTTCAG
pCA_ibpA_Y34R_Fw	GGCGGCcgtCCTCCGTATAACG
pCA_ibpA_Y34R_Rv	GGacgGCCGCCATTACTCTGGC
pCA_ibpA_Y34A_Fw	GGCGGCgctCCTCCGTATAACG
pCA_ibpA_Y34A_Rv	GGagcGCCGCCATTACTCTGGC
pCA_ibpA_Y34F_Fw	GCCAGAGTAATGGCGGCtttC
pCA_ibpA_Y34F_Rv	GTTCAACGTTATACGGAGGaaaGCCG
pCA_ibpA_Y34W_Fw	GCCAGAGTAATGGCGGCtggCC
pCA_ibpA_Y34W_Rv	GTTCAACGTTATACGGAGGccaGCCG
pCA_ibpA_Y34H_Fw	GAGCCAGAGTAATGGCGGCcatCC
pCA_ibpA_Y34H_Rv	GTTCAACGTTATACGGAGGatgGCCGC
